# Athérome coronaire éctasiant

**DOI:** 10.11604/pamj.2018.30.111.14916

**Published:** 2018-06-12

**Authors:** Abdelmajid Bouzerda, Laila Bendriss, Ali Khatouri

**Affiliations:** 1 Service de Cardiologie, Hôpital Militaire d’Instruction Mohamed V, Rabat, Université Cadi Ayyad, Faculté de Médecine et de Pharmacie, Marrakech, Maroc; 2 Service de Cardiologie, Hôpital Militaire Avicenne, Université Cadi Ayyad, Faculté de Médecine et de Pharmacie, Marrakech, Maroc

**Keywords:** Ectasie coronaire, thrombose, angioplastie coronaire, Coronary ectasia, thrombosis, coronary angioplasty

## Abstract

Les ectasies coronaires sont des pathologies relativement rares et mal connues. Leur étiologie chez l'adulte est le plus souvent athéromateuse. Ces anomalies exposeraient au risque de thrombose intracoronaire par stase sanguine, elles sont le plus souvent associées à des lésions sténosantes qui conditionnent le pronostic. Nous rapportons trois observations de patients hospitalisés pour un syndrome coronarien aigu ST positif avec un aspect de mégacoronaires noté à la coronarographie et nous discutons une revue de la littérature concernant ce type de lésions.

## Abstract (en)

Coronary ectasias are relatively rare and poorly known pathologies. In adults they are caused most often by atheromas. These anomalies can expose to intracoronary thrombotic risk due to blood stasis. They are most often associated with stenosing lesions which may affect the prognosis. We here report three observations of patients hospitalized for acute coronary syndrome associated with ST elevation and mega coronary appearance detected on coronarography and conduct a review of the literature on this type of lesions.

## Introduction

Le premier cas d'éctasie coronaire a été rapporté par Charles Bougon en 1812, cette entité reste encore mal connue et représente une forme particulière de la pathologie athéromateuse coronarienne. Le caractère thrombotique de ces lésions en l'absence de sténose coronaire associée été largement documenté par de petite séries et cas cliniques. Nous rapportons trois cas de syndromes coronariens aigus survenant sur des artères éctasiques et à la lumière de ces observations cliniques et une revue de la littérature nous discutons les particularités de cette pathologie.

## Patient et observation

**Figure 1:**
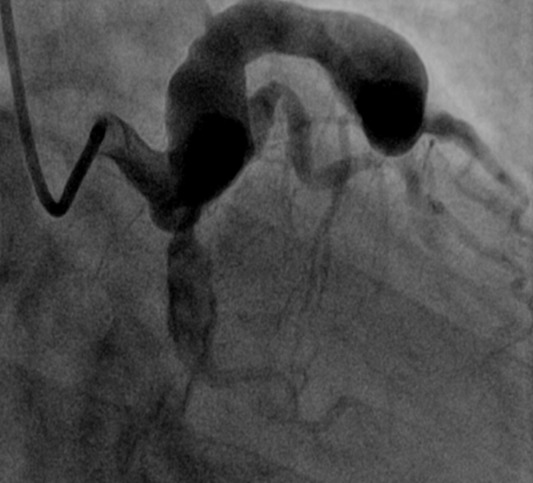
Incidence OAD caudale montrant un aspect de dolichomégacoronaire de l’artère interventriculaire antérieure

**Observation n 1:** Monsieur H.A âgé de 42 ans, est hospitalisé pour prise en charge d'un syndrome coronarien aigu ST positif de topographie antérieure thrombolysé à H4 avec succès par ténéctéplase. Ce patient présente comme facteurs de risque cardiovasculaires: un tabagisme non sevré évalué à 40 paquets-années et une hypertension artérielle sous Inhibiteur calcique. L'examen clinique est sans particularité. L'électrocardiogramme basal de repos montre un rythme régulier sinusal avec un aspect de sus décalage du segment ST en antérieur. L'échographie cardiaque montre une akinésie antérieure avec une fonction systolique ventriculaire gauche estimée à 40% par la méthode de simpson biplan. L'aorte ascendante n'est pas dilatée et il n'y a pas d'atteinte valvulaire mitroaortique. La coronarographie révèle un aspect de dolichomégacoronaire de l'artère interventriculaire antérieure responsable de l'infarctus (Figure 1), une sténose significative du segment proximal d'une artère circonflexe d'aspect thrombotique et éctasique, une mégacoronaire droite indemne de lésion sur l'ensemble de ces segments. Après discussion collégiale le patient a été mis sous traitement médical associant anti ischémiques, antiagrégants plaquettaires et anticoagulant avec évolution favorable.

**Figure 2:**
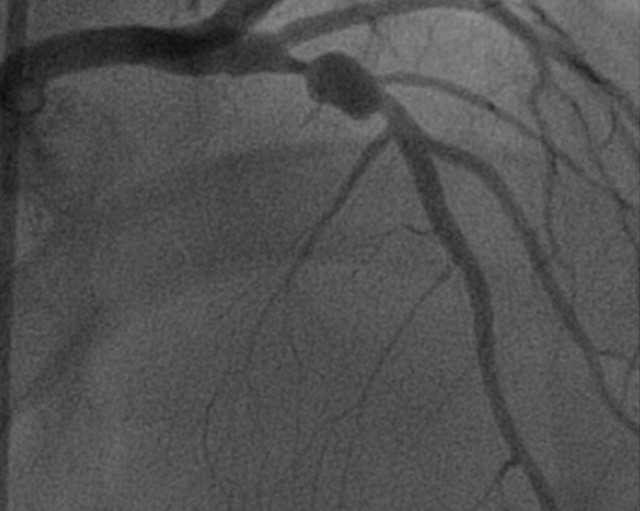
Incidence OAD craniale montrant une sténose subocclusive de l’artére interventriculaire antérieure avec un anévrysme poststénotique

**Observation n 2**: Monsieur M.M âgé de 47 ans, est hospitalisé dans les suites d'un syndrome coronarien aigu ST positif de topographie antérieure thrombolysé à H6 avec succès par Tenecteplase. Ce patient présente comme facteurs de risque cardiovasculaires un tabagisme non sevré évalué à 30 paquets-années et une hypertension artérielle bien équilibrée sous inhibiteur de l'enzyme de conversion de l'angiotensine. L'examen clinique est sans particularité. L'électrocardiogramme basal de repos montre un rythme régulier sinusal avec un aspect de nécrose en antéroseptal. L'échographie cardiaque montre une akinésie antérieure avec une fonction systolique ventriculaire gauche estimée à 45% par la méthode de simpson biplan. L'aorte ascendante n'est pas dilatée et il n'ya pasd' atteinte valvulaire mitroaortique. La coronarographie révèle une sténose subocclusive de l'artère interventriculaire antérieure (IVA) proximale avec un volumineux anévrysme post-sténotique (Figure 2), une artère circonflexe athéromateuse et infiltrée sans sténoses et une mégacoronaire droite siège des lésions étagées intermédiaires. Après discussion médicochirurgicale le patient a bénéficié d'une chirurgie de revascularisation coronaire avec un pontage mammaire interne gauche-Interventriculaire antérieur. Les suites postopératoires sont simples.

**Figure 3:**
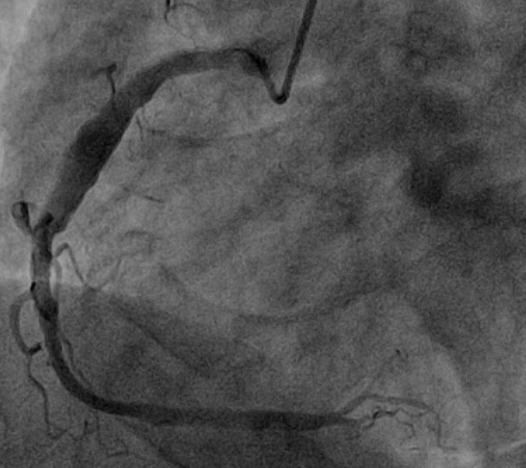
Incidence OAG montrant un aspect éctasique de l’artère coronaire droite

**Observation n 3**: Monsieur S.A âgé de 53 ans, est hospitalisé pour prise en charge d'un syndrome coronarien aigu ST positif de topographie inférieure non thrombolysé et non compliqué. Il présente comme facteurs de risque cardiovasculaires: un tabagisme ancien sevré, une hypertension artérielle sous Inhibiteur calcique et inhibiteur de l'enzyme de conversion de l'angiotensine. Ce patient a déjà fait l'objet d'une coronarographie pour un angor d'effort invalidant 03 ans avant cette admission montrant un aspect éctasique de l'artère coronaire droite sans sténose significative et un athérome modéré de l'artère interventriculaire antérieure et de l'artère circonflexe relevant d'un traitement médical. L'examen clinique à son admission est sans particularité. L'électrocardiogramme basal de repos montre un rythme régulier sinusal avec un aspect de sus décalé du segment ST en inférieur. L'échographie cardiaque montre une fonction systolique ventriculaire gauche estimée à 60% par la méthode de simpson biplan. Une cinétique segmentaire et globale conservée. L'aorte ascendante n'est pas dilatée et sans atteinte valvulaire mitroaortique. La coronarographie (Figure 3) révèle un aspect de coronaire droite éctasique avec un ralentissement du flux à ce niveau d'aspect thrombotique mais sans sténose significative, une plaque non significative de l'artère interventriculaire antérieure moyenne et une artère circonflexe indemne de lésion. Le patient a été mis sous traitement médical associant anti ischémiques, antiagrégants plaquettaires et anticoagulant avec une évolution favorable.

## Discussion

Les éctasies coronaires sont définis par une dilatation anormale d'une artère coronaire, intéressant plus de 50% de la longueur totale du vaisseau, ou focale, touchant moins de 50% de la longueur totale du vaisseau [**1**]. La prévalence globale de ces anomalies varie entre 0,2 et 6%, [2]. Elles sont plus importantes chez l'homme que la femme, respectivement de 2,2 et 0,5% [3], peuvent se voir à tout âge, L'athérosclérose demeure l'étiologie principale des éctasies coronaires chez l'adulte [4]. La maladie de Kawasaki en est toutefois une cause fréquente chez le sujet jeune [5]. Beaucoup plus rarement, ces éctasies coronaires peuvent être d'origine congénitale, ou auto-immune dans le cadre d'une vascularite [6]. Le sexe masculin serait un facteur majorant le risque d'ectasies coronaires en particulier pluritronculaire [2]. Le mode de révélation de ces anomalies coronaires est le plus souvent la découverte systématique lors d'un examen coronarographique chez un patient hospitalisé pour une urgence coronaire, pour un angor stable ou une ischémie myocardique silencieuse. Une forme de révélation doit être mentionnée: il s'agit des cas d'infarctus myocardique sans lésion significative retrouvée sur la coronarographie hormis l'éctasie coronaire. L'atteinte majoritaire de la coronaire droite est classique, dans sa partie proximale et moyenne, variant de 40 à 70% selon les séries rapportées. L'Ectasie coronaire expose au risque de thrombose par ralentissement du flux coronaire ce risque est bien corrélé à l'importance de l'éctasie, au risque de dissection et de rupture [1]. En Europe et Amérique du Nord, l'existence d'ectasie ne majore pas la mortalité dans les atteintes coronariennes athéromateuses, ne modifie pas le taux de survie des patients revascularisés chirurgicalement, ni celui des patients indemnes de sténose coronaire significative [7] certaines publications en Inde retrouvent une survenue comparable d'évènements cardiaques(décès, Syndrome coronarien aigu) dans les groupes ectasie sans sténose coronaire et sténoses coronaires sans ectasie et un pourcentage élevé d'infarctus du myocarde sur ectasies en l'absence de sténose coronaire significative [8]. L'existence l'athérome éctasiant pose des problèmes de prise en charge thérapeutique. Certains auteurs ont proposé l'instauration d'un traitement anticoagulant oral reposant sur des suppositions physiopathologiques et des cas cliniques. La revascularisation myocardique lorsqu'elle est effectuée est souvent chirurgicale (35-50% des cas) avec des résultats comparables entre athérome éctasiant et non éctasiant, pouvant associer réfection ou exclusion de la zone éctasique et pontage [9 ,10]. Quand l'angioplastie coronaire transluminale est indiquée les difficultés rencontrés sont essentiellement d'ordre technique avec notamment le choix et la taille du stent en raison de la disparité du calibre entre le segment sain et éctasique et la crainte de déployer le stent dans un segment fragile lorsque la lésion est incluse dans un segment anévrysmal L'emploi des stents couverts et auto-expansibles dans l'athérome éctasiant [11,12] est d'un grand secours dans la revascularisation de ces patients. L'amélioration du profil des ces endoprothèses devrait accroitre le pourcentage de revascularisation en cas athérome éctasiant.

## Conclusion

Les éctasies coronaires sont rares souvent associés à des sténoses et représente une forme particulière de l'athérome coronarien. Les difficultés techniques rencontrées au cours de l'angioplastie coronaire transluminale sont en rapport avec la complexité et les formes anatomiques particulières de ces lésions. Une utilisation répandue des stents couverts et autoexpandables pourraient améliorer la revascularisation de ces patients.
